# Trends in prevalence and correlates of intimate partner violence against women in Zimbabwe, 2005–2015

**DOI:** 10.1186/s12914-019-0220-8

**Published:** 2020-01-20

**Authors:** Jeanette Iman’ishimwe Mukamana, Pamela Machakanja, Nicholas Kofi Adjei

**Affiliations:** 1 Institute of Peace, Leadership and Governance, Africa University, Off Nyanga Road Fairview Road, P.O. Box 1320, Mutare, Zimbabwe; 2 College of Business, Peace, Leadership and Governance, Africa University, Mutare, Zimbabwe; 3 Health Sciences Bremen, University of Bremen, Bremen, Germany; 4 Leibniz Institute for Prevention Research & Epidemiology –BIPS, Bremen, Germany

**Keywords:** Intimate partner violence (IPV), Trends, Risk factors, Demographic and health surveys (DHS), Zimbabwe

## Abstract

**Background:**

Intimate partner violence (IPV) is a widespread problem affecting all cultures and socioeconomic groups. This study explored the trends in prevalence and risk factors associated with IPV among Zimbabwean women of reproductive age (15–49 years) from 2005 to 2015.

**Methods:**

Data from the 2005/2006, 2010/2011 and 2015 Zimbabwe Demographic and Health Survey (ZDHS) on 13,409 women (survey year: 2005/2006; *n* = 4081), (survey year: 2010/2011; *n* = 4411) and (survey year: 2015; *n* = 4917) were analyzed. Multiple logistic regressions and hierarchical modelling techniques were applied to examine the associations between demographic characteristics, socioeconomic status, media exposure and IPV against women. We further estimated IPV prevalence by type (physical, sexual and emotional) over time.

**Results:**

The prevalence of IPV decreased from 45.2% in 2005 to 40.9% in 2010, and then increased to 43.1% in 2015. Some of the risk factors associated with IPV were younger age, low economic status, cohabitation and rural residence. Educational attainment of women was however not significantly associated with IPV.

**Conclusions:**

The findings indicate that women of reproductive age are at high and increasing risk of physical and emotional violence. There is thus an urgent need for an integrated policy approach to address the rise of IPV related physical and emotional violence against women in Zimbabwe.

## Background

Intimate partner violence (IPV) refers to any assaultive and coercive behaviour that causes physical, psychological or sexual harm to a person in a relationship [1, 2]. IPV is pervasive globally [2–4], affecting all cultures and socioeconomic groups [5, 6]. Although this type of behavior can be perpetrated against men or women, evidence suggests that it is largely perpetrated by male partners against female partners of reproductive age [2, 7]. A recent multi-country study by the World Health Organisation (WHO) [2, 8] revealed that one out of three women experiences either physical or sexual violence in their lifetime worldwide. There are however regional variations, with the prevalence of IPV being observed to be higher in Africa (37%) and South-East Asia (38%) than in Europe (25%) and the Americas (30%) [2, 9].

IPV against women is a worldwide public health and human rights concern [10–12], as it has been shown to be a risk factor for various physical and mental health problems [7, 13–18]. Prior research has demonstrated that women who are sexually and physically abused by their intimate partners have a high risk of developing physical and mental health problems including traumatic stress, injury, depression infectious diseases such as Human Immunodeficiency Virus (HIV) and even death, compared to those not affected by IPV [7, 17–19].

The issue of IPV has become a global priority and there are efforts and high-level commitments towards addressing the issue. For instance, in an attempt to minimize or eradicate violence against women, the United Nations (UN) introduced conventions such as the convention on the Elimination of All Forms of Discrimination Against Women (CEDAW), among others that contain provisions to protect the rights and well-being of women to directly or indirectly curb the rising prevalence of violence against them [20, 21]. Furthermore, the Sustainable Development Goal five target two adopted by the UN in 2015 aims at ending all forms of violence against women [22, 23]. Moreover, various regions and nations have laws that criminalize intimate partner violence. For example, the African Charter on Human and People’s Rights (ACHPR)’S chapter 1, article 5, emphasizes that “*Every individual shall have the right to the respect of the dignity … … all forms of exploitation and degradation of man particularly slavery, torture, cruel, inhuman or degrading punishment and treatment shall be prohibited*” [24].

Despite the laws and legislations to protect women against violence, IPV is still on the rise in developing countries [25, 26]. In Africa, several factors including the patriarchy system, culture and social norms have been identified as contributing factors to the rise of IPV in the region [8, 26]. In general, IPV is tolerated and perceived as a cultural norm and accepted as a means to keep women disciplined and on track [4, 27–29]. In Sub Saharan Africa, over 75% of wife beating is justified, for example when a woman is deemed as not living up to her husband’s and society’s expectations [12, 26]. In some instances, local communities tolerate the male use of violence to maintain control over women [4, 26]. Thus, culture has been normalized and viewed as unavoidable in some communities over generations [4, 30, 31].

Furthermore, having a low economic status has been shown to increase women’s vulnerability to IPV [4, 32], because they might be financially dependent on their male intimate partners. As the average level of education of African women is usually lower than that of their male partners [33], they are more likely to be unaware of their rights and of the laws regarding IPV [34–38].

The relational approach theory suggests that differences in educational achievement, age, and carrier development may increase women’s vulnerability to IPV [8, 39]. In some circumstances, the financial situation of women may expose them to IPV, especially in conservative societies that usually stress normative roles of women [8, 25]. Conversely, some men may resort to violence to enhance their positions [28], especially where they feel powerless and threatened by their female partners’ socio-economic achievements [8, 25].

Over the past decades the media has become a critical tool in educating women on IPV in Sub-Saharan Africa [40], and has been utilized to prevent and respond to violence. Evidence suggests that the media is effective in raising awareness on IPV [41], and that it influences attitudes towards gender norms by alerting women and societies about human rights and violations of these rights [42].

### The case of Zimbabwe

The prevalence of IPV is high in Zimbabwe. According to data from a Demographic Health Survey (DHS) conducted in 2015, about 35% of women had experienced physical violence from the age of 15 and 14% had experienced sexual violence once in their lifetime. The report further revealed that 32% of married women had experienced spousal emotional violence [43]. Other studies further indicated that 40% of women and a third of men accepted and justified physical chastisement of women [44–46]. Although the Domestic Violence Act 14/2006 law exists in Zimbabwe, sexual offenses such as spousal rape, remain a widespread problem in the country [43, 44]. It has been reported that almost a quarter of married women who experience domestic violence also experience sexual violence [46]. Despite the government’s efforts to incorporate some of CEDAW’s protocols in the Domestic Violence Act, it has been noted that the government of Zimbabwe has not indorsed elective protocols [47] which are meant to address defilements comparable to individuals complaints procedures [48]. The domestication of these protocols has been hindered by poor implementation, administrative practices by both state and non-state institutions [47]. This has negatively impacted women and continues to place them in a subordinated state [49].

In 2010, the Media Monitoring Project Zimbabwe (MMPZ) assessed how Zimbabwe’s mainstream media fared in raising awareness of gender-based violence [50, 51]. Although the media is crucial in alerting communities and the authorities of these trending problems, MMPZ found that gender based violence only emerged as a secondary concern in the media [51]. In fact, it was observed that the media had not shown much inclination in raising awareness about gender based violence issues, even during the 16 days of activism against gender based violence campaign. Although, the media is a critical source of information on IPV issues [52, 53], studies on the influence and impact of the media on IPV are limited [12, 52–55].

As discussed previously, socioeconomic and cultural factors have been identified to be associated with IPV in some developing countries [4, 30, 33]. However, to the best of our knowledge, no study has examined the relationship between these factors and IPV in Zimbabwe, over time. The objective of this study is to explore the trends in prevalence and risk factors associated with IPV against women in Zimbabwe from 2005 to 2015. The following research questions will be addressed.

1. How do demographic characteristics and socioeconomic status (SES) of women influence IPV?
2. To what extent does media exposure of women impact their experience of IPV?

## Methods

The data for this study were from the 2005/2006, 2010/2011 and 2015 Zimbabwe Demographic and Health Surveys (ZDHS) [43]. The data are based on nationally representative surveys of men and women in their reproductive age that are undertaken by the Zimbabwe National Statistical Agency in collaboration with other international organizations. The ZDHS employed a two-stage stratified cluster sampling technique based on census enumeration areas (EAs) and household samples. The first stage was the selection of EAs in both rural and urban areas with probability proportional to the size, and the second stage involved household sampling. For this study, we limited our sample to currently married or cohabiting women aged 15–49 years. The samples for the final analyses after the exclusions were (survey year: 2005/2006; *n* = 4081), (survey year: 2010/2011; *n* = 4411) and (survey year: 2015; *n* = 4917).

### Measurement of the outcome variable

The outcome variable in this study was IPV. This variable was a combination of at least one type of intimate partner violence (physical, sexual or emotional) experienced by a woman. In the survey, the questions posed to women were for example “Did your husband or partner ever: slap you, push you, kick you, punch you or beat you up”? These questions were used to derive physical violence. Sexual violence was assessed by the questions: “Did your husband or partner ever: physically force you to have sexual intercourse with him even when you did not want to”? Or force you with threats to perform any sexual acts you did not want to? Further, women were asked whether their partner “said or did something to humiliate them in front of others,” “threaten to hurt or harm them” or “insult them to feel bad about themselves.” These set of questions were used to derive emotional violence. Responses were grouped and answers in the affirmative were categorized as ever experienced physical, sexual or emotional violence and coded “1”, while those who never experienced any form of intimate partner violence were categorized otherwise and coded “0”.

### Independent variables

The independent variables were group together into three broad categories: sociodemographic characteristics, socioeconomic status (SES) and exposure to media. SES was categorized using three measures: educational level (no formal education, primary, secondary or higher education), employment status (currently employed, not currently employed) and wealth index (poorest, poorer, middle and richer). The sociodemographic characteristics considered were age (15–19, 20–24, 25–29, 30–34, 35–39, 40+), marital status (married, cohabiting), number of children (no child, 1–2, 3–4, 5+), place of residence (rural or urban) and religious affiliations (Christians, Moslems, traditionalist, no religion). Exposure to media (newspaper, radio or television (TV)) was assessed in terms of frequency (no exposure, less than once a week, at least once a week). These explanatory variables were chosen to capture the individual and social context of IPV. All the variables were obtained from two types of questionnaires: the individual women’s and household questionnaire. The individual women’s questionnaire provided information on the women (i.e., demographic, socioeconomic and lifestyle characteristics) while the household questionnaire provided information on household possessions and amenities such as sanitation facilities, source of drinking water and household’s ownership of selected assets, which were used to create the “wealth index” [56].

### Statistical analysis

Descriptive and multiple regression analyses were performed in this study. In the first part of the analysis, percentages (%) were used to describe the prevalence and trends of IPV. Differences in IPV prevalence rates between the three survey years were examined using chi-square test. In the second part, binary logistic regression models were fitted to examine the associations between the independent variables and IPV using pooled data from 2005 to 2015. The binary logistic models estimate the likelihood of the outcome variable to be 1 (h = 1), and the conditional probability of experiencing the outcome (IPV) can be expressed mathematically as:

$$ pr\left(h=1|x\right)=\frac{\exp \left( x\beta \right)}{1+\exp \left( x\beta \right)} $$

The regression analysis was carried out using a three-step hierarchical modeling approach. This step-wise strategy allowed us to examine the independent impact of the groups of explanatory variables on the outcome variable. In the first model, logistic regression models were adjusted for sociodemographic characteristics. To examine the impact of women’s social status on IPV, SES variables were added in the second step (Model 2). Finally, media exposure variables were fitted in model 3. The prevalence of IPV and odds ratios with 95% confidence intervals (95% CI) was calculated using Stata Version 14 (Stata Corp, College Station, Texas, USA). All analysis were weighted to adjust for the DHS sampling design.

## Results

### Trends over time in the prevalence of intimate partner violence (physical, sexual or emotional)

**Fig. 1:**
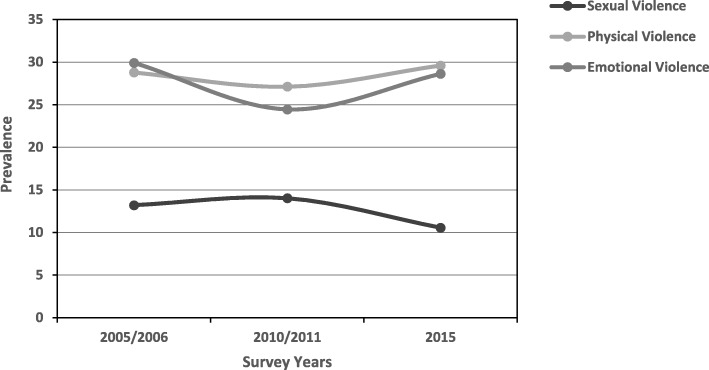
Prevalence of IPV among women of reproductive age (15–49 years) by survey year, Zimbabwe, pooled data, 2005–2015

**Fig. 2:**
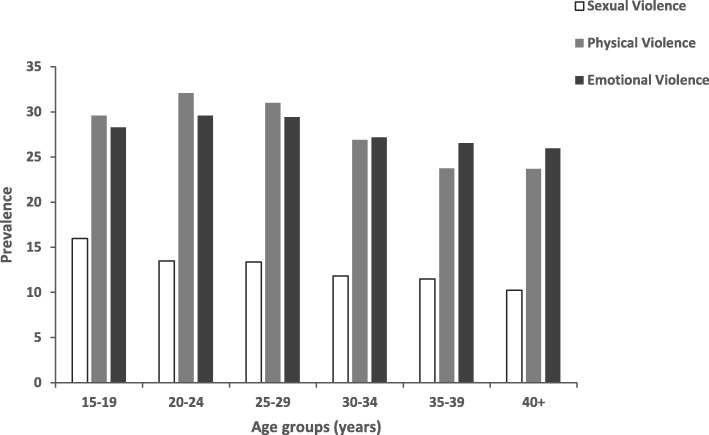
Prevalence of IPV among women of reproductive age (15–49 years) by age group, Zimbabwe, pooled data, 2005–2015

Table 1 presents the prevalence of IPV by sociodemographic characteristics, socioeconomic status (SES) and media exposure, from 2005 to 2015. Prevalence by age group and trends in prevalence of physical, sexual and emotional violence are shown in Figs. 1 and 2 respectively.

**Table 1:** Prevalence of IPV by sociodemographic characteristics, socioeconomic status and media exposure among women of reproductive age (15–49 years) by survey year, Zimbabwe, 2005–2015

Variables	2005/2006 (*n* = 4081)	2010/2011 (*n* = 4411)	2015 (*n* = 4917)
	IPV (%)	IPV (%)	IPV (%)
Age			
15–19	43.66	44.75	43.96
20–24	48.44	45.42	43.86
25–29	46.22	44.34	47.22
30–34	42.31	37.52	44.75
35–39	41.21	37.62	41.61
40+	46.15	34.10	36.05
Marital Status			
Married	45.20	40.66	42.67
Cohabiting	46.55	47.12	50.77
Number of children			
No child	33.12	35.69	34.81
1–2	45.93	41.56	42.60
3–4	44.80	41.59	44.27
5+	49.49	40.04	46.04
Place of residence			
Urban	38.33	37.28	42.61
Rural	48.14	42.64	43.43
Religion			
Christians	44.40	40.36	42.49
Moslems	42.86	45.64	49.65
Traditionalist	53.68	57.14	55.81
No Religion	49.64	44.83	54.68
Educational Level			
No education	50.76	40.16	47.46
Primary	46.47	45.23	45.78
Secondary and higher	43.92	38.75	41.96
Employment Status			
Not currently employed	41.18	38.73	40.67
Currently employed	51.87	44.99	46.35
Wealth (Index)			
Poorest	50.84	42.04	44.95
Poorer	51.71	47.67	44.63
Middle	43.99	40.92	44.38
Richer	32.63	32.30	38.10
Media exposure			
Newspaper			
No Media exposure	48.04	43.18	44.5
Less than once a week	42.88	38.30	41.1
At least once a week	38.50	34.26	41.5
Radio			
No Media exposure	47.99	41.07	41.13
Less than once a week	49.33	41.50	44.08
At least once a week	44.57	40.42	43.35
Television			
No Media exposure	48.65	41.99	43.35
Less than once a week	41.45	43.27	43.12
At least once a week	40.38	38.01	42.64
Total	45.23^a^	40.97^a^	43.10^a^

Note: ^a^ values with the same superscript are significantly different between the surveys at *p* < 0.05

The overall prevalence of IPV decreased from 45.2% in 2005/2006 to 40.9% in 2010/2011 (*P <* 0.001). However, in 2015, the prevalence increased marginally to 43.1% (*P <* 0.01) (Table 1). Regarding the various forms of IPV, the prevalence of sexual violence decreased from 13.2% in 2005 to 10.6% in 2015, but trends in physical and emotional violence showed a fairly similar pattern in 2005 (physical violence – 28.7%, emotional violence – 30.0%) and 2015 (physical violence – 29.5%, emotional violence – 29.6%) (Fig. 1).

In general, the prevalence of IPV increased with age to a maximum in the middle age (25–29 years), then decreased in the older age groups (35 years and above). While the prevalence of sexual and physical violence was higher among the younger age groups, emotional violence was generally similar across all age groups (Fig. 2).

The prevalence of IPV was generally higher among cohabiting than among married women. Whereas the prevalence among married women decreased between 2010 (45.2%) and 2015 (42.7%), it increased remarkably among cohabiting women over the same period from 46.6 to 50.7%. Regarding the number of children, the prevalence of IPV was higher among women with children and it increased as the number of children increased across all the survey years.

Further exploration with respect to the place of residence showed that women in the rural areas had a higher prevalence than those in urban areas. However, over the survey period, we observed an increase in the trend of the prevalence of IPV among women who lived in the urban areas, from 38.3% in 2005 to 43.0% in 2015, equalling that of women who lived in the rural areas in 2015 (43.0%). Regarding religion, the prevalence of IPV was higher among traditionalist women. Nonetheless, it decreased marginally from (57.1%) in 2010 to 2015 (55.8%).

By stratifying the data according to socioeconomic status (SES), we observed that over time, the prevalence of IPV was lower, among women with higher socioeconomic status (Table 1). Despite this finding, we observed an increase in the prevalence among this sub-group with higher SES, from 32.6% in 2005 to 38.1% in 2015. Meanwhile, the prevalence of IPV among the poorest decreased considerably from 50.8% in 2005 to 44.9% in 2015.

The trend analysis showed no consistent pattern in the prevalence of IPV over time, in terms of frequency of media exposure. However, we observed that the prevalence was relatively higher among women who had not been exposed to media (i.e. newspaper, radio or television).

## Logistic regression

The results of the adjusted odd ratio (OR) and 95% confidence intervals (CI) for the association between sociodemographic characteristics, SES, media exposure and IPV in a three step hierarchical model are shown in Table 2.

**Table 2:** Multivariate associations between sociodemographic characteristics, socioeconomic status, media exposure and IPV among women of reproductive age (15–49 years), Zimbabwe, pooled data from 2005 to 2015

Variables	Model 1^a^	Model 2^b^	Model 3^c^
	aOR (95% CI)	aOR (95% CI)	aOR (95% CI)
Age			
15–19 (ref)			
20–24	1.04 (0.88–1.2)	1.01 (0.85–1.1)	1.01 (0.85–1.20)
25–29	1.03 (0.87–1.23)	0.98 (0.82–1.17)	0.95 (0.84–1.07)
30–34	0.86 (0.71–1.03)	0.80 (0.67–0.97)**	0.76 (0.66–0.87)***
35–39	0.78 (0.64–0.95)**	0.72 (0.59–0.89)***	0.69 (0.59–0.80)***
40+	0.68 (0.55–0.83)***	0.63 (0.51–0.78)***	0.58 (0.49–0.68)***
Marital Status			
Married (ref)			
Cohabiting	1.32 (1.12–1.57)***	1.27 (1.07–1.50)**	1.26 (1.05–1.51)**
Number of children			
No child (ref)			
1–2	1.44 (1.28–1.68)***	1.43 (1.23–1.68)***	1.37 (1.23–1.68)***
3–4	1.61 (1.35–1.92)***	1.60 (1.35–1.91)***	1.59 (1.33–1.92)***
5+	1.93 (1.57–2.36)***	1.88 (1.54–2.31)***	1.93 (1.55–2.40)***
Place of residence			
Urban (ref)			
Rural	1.10 (1.02–1.18)**	0.87 (0.79–0.97)**	0.85 (0.76–0.96)**
Religion			
Christians (ref)			
Moslems	1.48 (0.90–2.43)	1.46 (0.90–2.43)	1.75 (0.98–3.14)*
Traditionalist	1.26 (0.95–1.67)	1.18 (0.89–1.58)	1.29 (0.95–1.76)
No Religion	1.24 (1.09–1.41)***	1.24 (1.08–1.41)***	1.24 (1.08–1.43)**
Educational Level			
No education (ref)			
Primary		1.10 (0.88–1.37)	0.96 (0.76–1.22)
Secondary and higher		0.96 (0.88–1.04)	0.92 (0.72–1.17)
Employment Status			
Not currently employed (ref)			
Currently employed		1.51 (1.40–1.63)***	1.50 (1.38–1.62)***
Wealth (Index)			
Poorest (ref)			
Poorer		1.10 (0.99–1.23)*	1.08 (0.96–1.21)
Middle		0.89 (0.80–0.98)**	0.88 (0.79–0.99)**
Richer		0.62 (0.53–0.72)***	0.64 (0.54–0.77)***
Media exposure			
Newspaper			
No exposure (ref)			
Less than once a week			0.94 (0.85–1.04)
At least once a week			0.95 (0.82–1.08)
Radio			
No exposure (ref)			
Less than once a week			1.06 (0.95–1.16)
At least once a week			1.07 (0.98–1.18)
Television			
No exposure (ref)			
Less than once a week			1.02 (0.89–1.16)
At least once a week			1.03 (0.90–1.15)
Observations	13,409	13,409	13,409
Pseudo R2	0.0143	0.0237	0.0341
Log Likelihood	− 9033.5425	− 8946.9156	− 7871.9548

*Notes*: aOR- adjusted Odd Ratio, *** *p* < 0.001, ** *p* < 0.01, * *p* < 0.05. Regression includes age at first cohabitation

^a^Includes sociodemographic characteristics variables

^b^Includes sociodemographic and socioeconomic status (SES) variables

^c^Includes sociodemographic, socioeconomic status (SES) and media exposure variables

In model 1, based solely on sociodemographic characteristics of women, an association between women’s age and IPV was observed. Older women (40+ years) were less likely to experience IPV (aOR = 0.68; 95% CI = 0.55–0.83) compared to younger women (15–19) years. We also found marital status to be strongly associated with IPV, whereby cohabiting women had higher odds of reporting IPV (aOR = 1.32; 95% CI = 1.12–1.57) compared to married women. The number of live children of women was significantly associated with IPV. Women who had more children (e.g. 3–4) were more likely to experience IPV (aOR = 1.61; 95% CI = 1.35–1.92) compared to their counterparts without children. Religion was also strongly associated with IPV, with women with no religious affiliation having a higher likelihood of reporting IPV (aOR = 1.24; 95% CI = 1.09–1.41) compared to Christians. The regression analysis also confirmed the role of geographical area or location. Considering only sociodemographic characteristics of women, we observed that women living in the rural areas were about 10% more likely to be report IPV (aOR = 1.10; 95% CI = 1.02–1.18) compared to their counterparts in the urban areas.

With the inclusion of SES in model 2, all the sociodemographic characteristics of women maintained their significant influence on the experience of IPV, except place of residence. Adjusting for SES attenuated the association between the place of residence of women and IPV, whereby women living in the rural areas were now less likely to report IPV (aOR = 0.87; 95% CI = 0.79–0.97) compared to their counterparts in the urban areas. In the model, wealth and employment status of women showed significant effects on the likelihood of experiencing IPV. Richer women had lower odds of reporting IPV (aOR = 0.62; 95% CI = 0.53–0.72) compared to the poorest. We also observed a strong association between employment status and IPV, with women who were employed being about 50% more likely to report IPV (aOR = 1.51; 95% CI = 1.40–1.63) compared to those not employed. Educational attainment of women was however not significantly associated with IPV in both model 1 and 2. When media exposure of woman was introduced in the full model (Model 3), we observed a significant reduction in the effect sizes of sociodemographic characteristics on IPV. However, media exposure (i.e. newspaper, radio or television) of women was not significantly associated with IPV.

## Discussion

IPV against women has not only been widely investigated in the extant literature, it has also drawn much attention in state organisations as well as in the international community. Nonetheless, this is the first study to examine the trends in the prevalence and risk factors of IPV against women in Zimbabwe, using DHS data collected from 2005 to 2015. Overall, the results revealed that the prevalence of IPV decreased from 45.2% in 2005 to 40.9% in 2010, and then increased again to 43.1% in 2015. Regarding the various forms of IPV, the prevalence of emotional violence, the most popular form of IPV against women in Zimbabwe and other Sub Saharan African countries [57] was generally similar across all age groups. The results further showed that age was inversely associated with increased experience and vulnerability to IPV, with the younger age groups being more affected than the older ones. This pattern is consistent with other previous studies [45, 58–60] that found IPV to be higher among younger adults because they are likely to engage in aggressive and violent behaviours [45, 58]. Young women who are in unions may be vulnerable to IPV due to lack of educational opportunities, power inequalities [61–63] and they are more likely to depend on their partners [64–66] than older women. This may limit their autonomy in unions [61], and their lack of autonomy may encourage and increase control from their partners.

The findings revealed a pattern where the prevalence of IPV increased as the number of children increased. A possible explanation for this phenomenon is that women may not want to leave their matrimonial homes as they may tend to secure the welfare of their children as well as fear of losing offspring [67]. On the other hand, pregnancy has been shown to increase the risk of IPV [68–70], and this phenomenon has been attributed to economic dependency of women [68, 71, 72]. The results are also in line with those studies which concluded that the prevalence of IPV was higher among cohabiting women as compared to married women [73]. Our findings that the prevalence of IPV was higher among women in the rural areas and among traditionalist probably has to do with the perception that the traditionalist, whose proportion is higher in rural areas [74], are deeply rooted in culture and stress the issue of traditional beliefs that justify male dominance and abusive acts [27, 45]. Furthermore, dominant traditional femininity practices that encourage hegemonic masculinities, characterised by women’s subordination and accommodation of men’s interest may encourage and justify violence against women [75–77].

Surprisingly, we did not find any significant association between education level and IPV. While our findings are consistent with some prior studies [8], other studies found education to be protective of IPV [78, 79], as it reduces acceptance of wife beating and other forms of abuse [80]. The findings of two other studies clearly demonstrate the controversy related to this aspect. Whereas Lawoko and colleagues [81] found education to be crucial in reducing the risks of experiencing IPV among Kenyan women, Taillieu and Brownridge [82] observed that higher education attainment of women increased the risk of experiencing IPV. The controversy might arise from the fact that having a higher education may be related to political knowledge and participation in decision making [83], thereby enabling women to challenge abusive practices and make decisions. This act may eventually threaten the superiority of men, who in turn might resort to the use of coercive power to protect their identity. In other studies, women with low levels of education were found to be more likely to experience sexual violence [12, 31, 84]. Despite these mixed findings, we speculate that empowerment of women through education does not shield them from being abused.

Regarding economic status, the results showed that wealth and employment status of women had potential impacts on IPV, which is consistent with findings of previous studies [39, 84, 85]. The observation that employed women were more likely to report IPV than their unemployed counterparts has been linked to the view that employed women devote less time to traditionally prescribed roles such as household chores [8, 86], which may result in spousal conflict [86–88]. The employment status of a woman might also threaten the partner’s status and role as the family breadwinner, especially in conservative communities that stress gender roles. Being employed has however also been said to reduce IPV, as it empowers women economically, enabling them to acquire wealth (resources) and reducing dependence on their partners [89].

We also found that women who were rich had lower odds of experiencing IPV compared to poor women. This may be as a result of empowerment driven from wealth, which eventually reduces their dependence on their partners [32, 81]. Previous studies have also noted that the financial status of a woman may be a protective measure against IPV [30–32, 39, 81]. Poverty, on the other hand, has a great potential of exposing women to IPV [90–92], as poor women heavily depend on their partners and may not be in a position to bargain [32, 90, 91]. Conversely, as with education and employment, the wealth of a woman may put her at risk of being abused, because having more financial resources than a partner may create inconsistencies of status within the family. Some studies showed that men feel threatened by the wealth of their partners and thus secure themselves through violence [39, 81, 92–94]. This can be explained by the association between gender roles and the perpetration of IPV. Previous studies have indicated that gender roles are socially constructed as the product of gender system that perpetuates gender disparities, which may increase male dominance and women subordination [95–98]. This phenomenon may encourage violence, and some men portray these expected roles through violence [95, 99].

Concerning media exposure, the findings indicate that a significant number of women have limited access to media. Even though the prevalence of IPV was higher among women who did not have access to any form of media (TV, radio and newspaper), we did not find any significant association between women’s exposure to media and IPV when other social and demographic factors were considered. This phenomenon may be explained by the little attention given to issues of IPV by the media in Zimbabwe and Africa as a whole. Findings of a previous study by Lawoko and colleagues [81] in Kenya indicated that issues of domestic violence and empowerment receive little attention in the local media. Further, the media generally projects IPV as a private issue that should be solved by the affected individual [40].

## Strengths and limitations

Our study has both limitations and strengths. One limitation is that the data from the DHS may underestimate the prevalence and extent of IPV due to underreporting, respectively social desirability [100, 101]. Further, the cross-sectional nature of the study design does not permit fortitude of causality between variables. While the independent variables can cause the outcome variable, the outcome variable can also be a risk factor. Thus, there might be a reciprocal relationship between the dependant variable and the independent variables. This study is delimitated to women who are either married or cohabiting. Hence, there is the need for further research into other forms of intimate relationships. Nonetheless, using DHS data has several advantages because the selected participants were sampled using probability sampling methods. In addition, the interviewers were well trained and supervised to adhere to the WHO regulations or guidelines [102, 103] that ensure the safe collection of data on domestic violence without causing harm.

## Conclusion

Our study provides the first evidence of the trends in prevalence and correlates of IPV against women in Zimbabwe. The findings indicate that women of reproductive age are at high and increasing risk of IPV and there is an urgent need for an integrated policy approach to address the rise of physical and emotional violence against women by their intimate partners in Zimbabwe.

## Data Availability

The data used for this study comes from the Demographic and Health Survey (DHS). Detailed information on the survey design and characteristics are provided on the DHS homepage, https://dhsprogram.com/Data/

## Abbreviations

ACHPR: African Charter on Human and People’s Rights
CI: Confidence Intervals
DHS: Demographic Health Survey
EAs: Enumeration Areas
HIV: Human Immunodeficiency Virus
IPV: Intimate partner violence
MMPZ: Media Monitoring Project Zimbabwe
OR: Odd Ratio
SES: Socioeconomic Status
TV: Television
UN: United Nations
WHO: World Health Organisation
ZDHS: Zimbabwe Demographic and Health Survey
